# Impact of Congenital Visual Impairment on Early‐Life Exploration: Behavioral Analysis of Temporal and Motor Parameters During a Reach‐to‐Grasp Playful Task

**DOI:** 10.1111/desc.70067

**Published:** 2025-08-24

**Authors:** Petri Stefania, Riberto Martina, Setti Walter, Campus Claudio, Vitali Helene, Signorini Sabrina, Tinelli Francesca, Serafino Massimiliano, Strazzer Sandra, Giammari Giuseppina, Cocchi Elena, Gori Monica

**Affiliations:** ^1^ U‐VIP Unit for Visually Impaired People Italian Institute of Technology Genoa Italy; ^2^ Department of Informatics Bioengineering, Robotics, Systems Engineering (DIBRIS) University of Genoa Genoa Italy; ^3^ Developmental Neuro‐Ophthalmology Unit IRCCS Mondino Foundation Pavia Italy; ^4^ Department of Developmental Neuroscience IRCCS Fondazione Stella Maris Pisa Italy; ^5^ Department of Surgical Science Division of Ophthalmology IRCCS Institute Giannina Gaslini Genoa Italy; ^6^ IRCCS Scientific Institute E.Medea Bosisio Parini Lecco Italy; ^7^ Rehabilitation Unit for Visually Impaired Children, David Chiossone Foundation Genoa Italy

**Keywords:** bimanuality, body‐midline crossing, development, early life stages, reach‐to‐grasp behavior, visual impairment

## Abstract

Reach‐to‐grasp behavior is a key developmental milestone in infants, involving coordinated actions such as arm transport, hand pre‐shaping, and hand opening and closing. Vision guides the development of these skills, and delays in visual input can impact infants with early visual impairments. However, the effects of a congenital visual impairment on reach‐to‐grasp behavior in early life remain largely unexplored. To address this gap, we compared the reach‐to‐grasp abilities of sighted (S) and visually impaired (VI) infants and children, focusing on temporal (Movement and Pick‐up times) and motor parameters (body midline crossing with one or two hands and hand preference). We hypothesized that VI children would face greater challenges in planning and executing a rapid and accurate reach‐to‐grasp movement, particularly during the pick‐up phase. To investigate this, we asked sighted and VI infants and children to grasp black spheres of different sizes, placed centrally, on the right, or the left of a table in a dimly lit room. Three key findings emerged from our analysis. First, VI children required more time to pick up the spheres compared to their sighted peers. Second, VI children showed a reduced frequency of one‐handed body midline crossing when reaching for lateral spheres, but showed an age‐related increase, especially when using both hands. Third, VI children showed no hand preference, unlike S children who favored their right hand for crossing the body midline. These results highlight the role of visual experience in developing effective goal‐directed movements and support creating early evidence‐based rehabilitation procedures. A video abstract of this article can be viewed at https://youtu.be/bjwkMQmdFoE.

**Summary:**

- Understanding the impact of visual impairment on exploration abilities is crucial, especially in early developmental stages.
- Currently, there is a significant research gap concerning fine motor skills, particularly reaching and grasping, in visually impaired infants during development.
- We proposed a playful task to collect behavioral data on reaching and grasping skills in visually impaired children.
- Results shed light on the pivotal role of visual experience during the first years of life in shaping the maturation of reaching and grasping skills.

## 1 Introduction

The ability to act within the environment is a fundamental milestone for the child's development from the earliest stages of life. Planning actions with specific goals, for example, reaching and grasping an object, is crucial to promote a functional exploration of the environment and fostering learning about the surrounding world (Bruner and Koslowski 1972). The development of these motor strategies in the early months of life supports the emergence of more efficient and complex manual skills (e.g., shaking, hitting, throwing) and of some cognitive functions, such as concept formation, symbolic thinking, and language skills (Piaget 1952). This requires the ability to integrate somatosensory and visual cues with the sensory‐motor memory system and the use of anticipatory strategies to optimize movement execution (Camponogara and Volcic 2021; Johnasson and Cole 1992).

According to previous studies, reach‐to‐grasp skills typically develop around 3 to 6 months (Hadders‐Algra 2013). From the first months of life, infants show motor strategies tailored to the object's characteristics, such as the differentiation in the use of one or two hands, and the hand's anticipatory shaping to align with the features of the object. However, given the challenges in designing suitable experimental paradigms for testing infants at such a critical age, the study of the development of reach‐to‐grasp behavior remains an ongoing area of research with mixed findings. For instance, Fagard et al. (2000) pointed out that the ability to reach and grasp an object with one or two hands according to object size typically develops around the first year of life (Fagard 2000). However, other studies found that even at earlier stages infants tend to use both hands without strategically selecting the most efficient motor solution based on the object's characteristics (Newell et al. 1989; Rocha et al. 2013; Van Wermeskerken et al. 2011). A high variability about the critical developmental period of reach‐to‐grasp ability was also demonstrated, showing that it is still evolving well beyond age 5 years (Olivier et al. 2007; Zoia et al. 2006). For example, Olivier et al. (2007) observed high variability at the age of 6 years, followed by transitional coordination at age 8, with the adult‐like integration of reaching and grasping still incomplete even at age 11. This developmental process, which involves increased coordination over time, heavily relies on automatic sensory control (Gaveau et al. 2014).

Analogous to other developmental milestones in prehension, the acquisition of the ability to cross the body midline to reach and grasp objects placed on sides occurs approximately between 4.5 and 7 months (Morange and Bloch 1996). This ability is related to the development of bimanual reach‐to‐grasp actions, particularly when the child needs to use both hands according to the size of an object placed on the side to effectively grasp it (van Hof et al. 2002).

In addition, the development of a hand preference, even for simple actions such as reaching and grasping for an object, supports the acquisition of more complex manual capabilities (e.g., manipulation of multiple objects) (Hinojosa et al. 2003), which is essential for the development of various adaptive functions, including the execution of coordinated bimanual actions (Michel et al. 2016). Several studies highlighted that the majority of adults are right‐handers, a trend likely driven by an interplay of neuromotor asymmetries and postural preferences that are already present in utero (MacNeilage et al. 2009). In contrast, studies on infants revealed greater variability in handedness, suggesting that the development of hand preference is shaped over time by a dynamic interplay of genetic and environmental factors (Fagard 2013; Marcinowski et al. 2024).

Few studies offered a thorough perspective on the contributions of multisensory processing to reach and grasp abilities showing a right‐hand/left‐hemisphere specialization for visually guided tasks and left‐hand/right‐hemisphere specialization for haptically guided object recognition (Gonzalez et al. 2007; Stone and Gonzalez 2015). This suggests that information from different senses is fundamental for motor development and adaptive functioning, with vision as the dominant sense for the maturation of skills required for spatial interaction and object manipulation (Braddick and Atkinson 2011; Bremner et al. 2008; Hood and Atkinson 2008; Tadić et al. 2009; Tröster and Brambring 1993). For instance, visual impairment during childhood can significantly alter these processes, leading to delays in fundamental motor milestones, such as self‐initiated posture and locomotion, as well as compromised spatial and sensory‐motor skills (Cappagli and Gori 2016; Esposito et al. 2021; Gori et al. 2014, 2021; Prechtl et al. 2001; Troster et al. 1994). This disruption likely stems from the primary role of vision in cross‐sensory calibration during body representation and space perception (Dale and Sonksen 2002; Fazzi et al. 2011; Gori et al. 2012, 2021; Purpura and Tinelli 2020).

Most of the studies that examined reach‐to‐grasp skills in participants with atypical development, have focused on children with hemiplegic cerebral palsy who exhibit a notable impairment in coordinating force during object picking‐up and release actions (Eliasson and Gordon 2000; Mackenzie et al. 2009) or individuals with autism spectrum disorder, who demonstrated compromised motor performance in reach‐to‐grasp movements due to difficulties in motor planning (Rodgers et al. 2019; Sacrey et al. 2018).

However, few studies explored reach‐to‐grasp skills in visually impaired participants. These primarily focused on adults and children older than 3 years, particularly those with visual impairment due to cerebral damage (Pardhan et al. 2011; Rossit et al. 2011). For example, Ittyerah et al. (2000, 2009) evaluated children aged from 6 to 15 years with congenital blindness through diverse manual tasks (e.g., cutting paper with a pair of scissors, drawing a line, throwing a ball), and observed that both congenitally blind and blindfolded sighted children displayed a preference for using their right hand (Ittyerah 2000, 2009). A recent study explored how early visual experience shapes the development of action and perception prediction during a reach‐to‐grasp task in a sample aged 8–21 years who had surgery for congenital cataracts. They focused on both temporal and kinematics parameters of reach‐to‐grasp behavior, dividing the entire action into two components: the transport component, referred to as “Movement”, and the grip component, referred to as “Pick‐up.” They discovered that early visual deprivation affects the ability to use visually estimated object features during grasping, even after cataract removal. Specifically, in cataract‐treated patients, while both temporal parameters of reach‐to‐grasp decrease after about 1 year following vision restoration, the kinematic parameters of grasping (e.g., grip scaling and grip force) continue to differ from sighted peers (Piller et al. 2023). In other words, they suggested that the lack of visual experience from birth impacts the ability to plan and pre‐adapt their hands to grasp objects effectively. These findings further highlight the fundamental role of early visual experience in shaping the development of goal‐directed motor abilities. Particularly, they emphasize how early visual input is essential for programming motor actions that enable children to interact more effectively with the surrounding environment and the objects within it. However, despite the relevance of early visual experience on the development of reach‐to‐grasp skill, to our knowledge, no studies have been conducted on children from the first year of age with congenital visual impairment resulting from damage to the peripheral visual pathways.

### 1.1 The Current Study

To fill this gap, we investigated the contribution of early visual experiences on the development of reach‐to‐grasp skills in a cross‐sectional study. Specifically, we asked congenitally visually impaired (VI) and sighted (S) children, with an age range between 0 and 6 years, to reach and grasp spheres of different dimensions and return them to the experimenter. In line with Piller's study (Piller et al. 2023), we analyzed time (i.e., the duration of the reach‐to‐grasp action) and, in line with Van Hof's study (van Hof et al. 2002) some motor (i.e., one and two‐hand body midline crossing and hand preference during reach‐to‐grasp for the lateral spheres) parameters to study differences in reach‐to‐grasp abilities between S and VI children. We also considered the effect of age on the development of these skills within each group.

We predicted that S children are faster in Pick‐up than VI children. We also hypothesized (i) a reduced tendency to cross the body midline during reach‐to‐grasp for lateral spheres in VI children, and that (ii) VI children would not show any hand preference when reaching for spheres placed laterally. We also expected (i) a reduction in Movement time with age in both groups and (ii) an increase in the tendency to cross the body midline with age in VI children, particularly when using both hands to reach lateral spheres.

## 2 Methods

### 2.1 Participants

Thirty‐two S children (18 females, age range: 1.0–5.8 y.o, mean age: 3.3 ± 1.3 y.o) and fifteen VI children (5 females, age range: 0.7–6.0 y.o, mean age: 2.5 ± 1.5 y.o.) participated in the experiment. The experimental procedure took place in Genova, Pavia, and Calambrone (Italy). The sample was drawn from an urban environment (Genova and Pavia) and a suburban environment (Calambrone). Participants were Italian citizens from the general population, with a medium to high socioeconomic status.

VI children were classified according to vicarious diagnostic normative criteria with congenital visual impairment from damage to the peripheral visual pathways with residual vision comprised between 1.3 and 0.7 LogMAR (see Table S1). The exclusion criteria were: history of prenatal infections, fetal distress during delivery, other perinatal distress or metabolic disorders, motor or cognitive impairment, and neurological disorders. The legal guardians of the participants signed the written informed consent. This study was approved by the Ethics Committees of centers participating in the study (Tuscany Region Ethics Committee—Paediatric, Liguria Region Ethics Committee, IRCCS E.Medea Ethics Committee, IRCCS Mondino Foundation Ethics Committee).

### 2.2 Experimental Procedure

**FIGURE 1:**
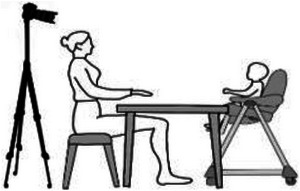
Illustration of the experimental setup: the experimenter sitting across from the child, both positioned in front of a table. The camera is placed on a tripod, directed toward the participant as they perform the reach‐to‐grasp task.

Before starting the experiment, the child was seated in a small comfortable chair, which provided postural support, and allowed its upper limbs to move freely and to lean against the backrest. The experiment was conducted in a dimly lit room, with the child's behavior recorded by a digital video camera in night mode. The camera was positioned 80 cm in front of the table, facing the infant's frontal midline, at a height of 120 cm and a 45° angle to their frontal plane (Figure 1). Video data were recorded at 25 frames per second for offline coding. Before presenting the stimuli, we measured the length of the child's arm and the distance between his shoulders to place on the table the markers which indicated the position for the stimuli (i.e., in front of the child's left and right shoulders, and at the body midline).

**FIGURE 2:**
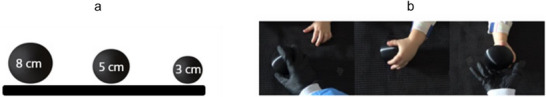
Experimental stimuli. (a) Black spheres with different diameter sizes used in the experiment: 3, 5, and 8 cm; (b) Video‐frames showing the reach‐to‐grasp task.

The stimulus set consisted of three black spheres fabricated in Acrylonitrile Butadiene Styrene (ABS) material by using Stratasys Connex 500 multi‐material 3D printer. We used a small, medium, and big sphere that varied in diameter (3, 5, and 8 cm, respectively) (Figure 2a). To investigate whether the dimension of the stimuli could affect motor strategies, we conducted a second exploratory experiment involving a subsample of the participants, using two additional spheres with increased diameters (see Figure S1). The color of the spheres and the gloves worn by the experimenter were deliberately selected to minimize contrast with the black table in front of which children were seated. This was done to reduce visual stimulation, even for sighted children who may not maintain continuous eye contact throughout the experiment (Figure 2b).

We instructed the children to reach and pick up the sphere placed on the table and then to pass it back to the experimenter who sat in front of them. We provided the children with information on the experimenter's hand position by tapping the back of his hand twice against the table, and the sphere's position by striking the ball twice on the table. To present the stimuli, we randomized the dimensions (e.g., small, medium, big) and the positions (e.g., left, right, center). This order was the same for each participant (see Table S2). No time constraint was imposed for reaching and grasping the sphere. Each trial ended successfully when the child returned the sphere to the experimenter within 10 s of grasping it, otherwise, we moved on to the subsequent trial. The total duration of the experiment was approximately 10 min (nine trials in total).

### 2.3 Observational Coding and Data Analysis

#### 2.3.1 Observational Coding

Reach‐to‐grasp behavior parameters were extracted from the video records by two naïve raters. The raters were trained by the authors involved in the data collection using sample videos. The raters not involved in the study and were blind to its purpose. This approach was deliberately chosen to minimize potential biases on parameter extraction (e.g., awareness of the study's goal). The raters examined both time and motor parameters for each trial independently, frame by frame, using Avidemux (*Avidemux—Main Page*, n.d.) an open‐source software application for non‐linear video editing and transcoding multimedia files. We included only successful pick‐up trials both in the time and motor parameters analyses. For completeness, we reported the number of attempts required for a successful pick‐up in both groups in the Supplementary Materials (Tables S3 and S4; exploratory experiment: Tables S5 and S6), based on raters agreement.

In line with previous study (Piller et al. 2023), the time parameter, that is, the duration (in seconds) of reach‐to‐grasp action, was divided into: (i) *Movement time*, the time interval between the initiation of upper limb movement toward the target and the first touch of the sphere with one or both hands; (ii) *Pick‐up time*, is the time interval between the initial touch of the sphere with the hand and the actual grasp of it. These time intervals were extracted from the video analysis independently by each rater. Subsequently, the intervals recorded by the raters were averaged for each trial. We calculated the Intraclass Correlation Coefficient (ICC) using a two‐way random‐effects model to assess the inter‐rater reliability of Movement Time and Pick‐up time across all trials. The results indicated good reliability for both measures: Movement time ICC = 0.70, 95% CI [0.65, 0.75], *F*(405,405) = 5.62, *p* < 0.001; Pick‐up time ICC = 0.66, 95% CI [0.60,0.71], *F*(405,405) = 4.87, *p* < 0.001.

We also defined the motor parameters of the trials involving spheres placed on sides as the number of times the child picked‐up the sphere using one hand *(one‐hand body midline crossing)* and both hands *(two‐hands body midline crossing)*. Specifically, for each trial, the raters assigned a score of 0 when the child did not cross the body midline, and a score of 1 when he did with one‐hand (for one‐hand body midline crossing) and with both hands (for two‐hands body midline crossing). We included in the analyses only the trials wherein the raters were in agreement, meaning they both recorded the same observation. Then, we calculated the percentage of body midline crossing, by dividing the sum of trials when the child crossed the body midline out of the total number of trials with lateral spheres, separately for each motor‐ parameter (i.e., one and two‐hands body midline crossing). We also evaluated the *hand preference*, that is, the hand used to reach and grasp the sphere on the sides. For each participant, we calculated the percentage of body midline crossing performed with the right and the left hand out of the total trials with the spheres on the sides.

#### 2.3.2 Data Analysis

All analyses included in this study were carried out using RStudio 4.2.2 software (R Core Team 2022).

We used Linear Mixed Models (LMMs) to analyze time parameters and hand preferences. Model fitting was undertaken using the *lmer* function of the lme4 package (Bates et al. 2015). The LMM predictors were evaluated using Type II Wald *X* ^2^ tests as implemented in the Anova function of the car package (Fox and Weisberg 2019). Significant fixed effect were further investigated with the *emmeans* function of the emmeans package by obtaining estimated marginal means and computing their contrasts (Lenth 2024). In addition, we explored whether the age‐related change in performance differed between groups using the *emtrends* function of the emmeans package, to estimate and compare the slopes of age within each group.

Then, we used Linear Model (LM) to analyze motor parameters (one and two‐hands body midline crossing). Model fitting was performed using the *lm* function, which is part of the base R package. The statistical significance of the predictors was evaluated using Type II Wald *X* ^2^ tests. Significant fixed effects were further investigated with the *emmeans* function. We also explored whether the age‐related change in performance differed between groups using the *emtrends* function.

We first investigated differences between groups in time and motor parameters, separately. For time parameters, we entered the Time (in seconds) as dependent variable, type of Action (Movement and Pick‐up) and Group (VI and S) as categorical predictors, and participant ID as a random effect (Time ∼ Action * Group + (1|ID)). For motor parameters (one and two‐hands body midline crossing) we performed two separate LM. In the first model, we entered the percentage of one‐hand body midline crossing as the dependent variable, while in the second model, the dependent variable was the two‐hands body midline crossing parameters. In both models, Group (VI and S) was used as categorical predictor.

Then, we explored how these parameters change with age. Specifically, for time parameters, we entered the Time (in seconds) as the dependent variable, Group (VI and S) and Age (in years) as predictors, and participant ID as a random effect into two separate LMMs, one for Movement and one for Pick‐up, (Movement time ∼ Group * Age + (1 | ID); Pick‐up time ∼ Group * Age + (1 |ID)).

For motor parameters, we entered the percentage of one‐hand body midline crossing as dependent variable, and Group and Age as predictors (one‐hand body midline crossing % ∼ Group * Age). The same approach was used with the two‐hands body midline crossing parameter (two‐hands body midline crossing % ∼ Group * Age). Previous studies suggested gender differences in reach‐to‐grasp behavior (Copley‐Mills et al. 2016; Zheng et al. 2022). Although we did not match the groups for Biological Sex for practical challenges during the recruitment of children, especially from clinical population, we also tested extended models including Biological Sex as a fixed effect, for each body midline crossing variables. We compared the model fit using the Akaike Information Criterion (AIC), which demonstrated that including Biological Sex in our study did not improve model fit. Therefore we took into account the model without this fixed effect for the main analyses. These results are reported in Supplementary Materials (*Results: effect of Biological sex on body midline crossing*). Finally, we explored any hand preference during one‐hand body midline crossing within each group. We conducted an LMM with the percentage of one‐hand body midline crossing as dependent variable, Hand (left and right) and Age as predictors, and participant ID as random effects (one‐hand body midline crossing % ∼ Hand * Age + (1 | ID)), as the analysis was performed within each group, with two observations per subject: one percentage of body midline crossing for the right and one for the left hand.

We did not include sphere position and size as fixed effects in the analyses for maximizing statistical power.

Bonferroni post hoc corrections for multiple comparisons (*p* < 0.05) were used to explore the nature of the effects.

## 3 Results

### 3.1 Increased Pick‐up Time in Visually Impaired Children

**FIGURE 3:**
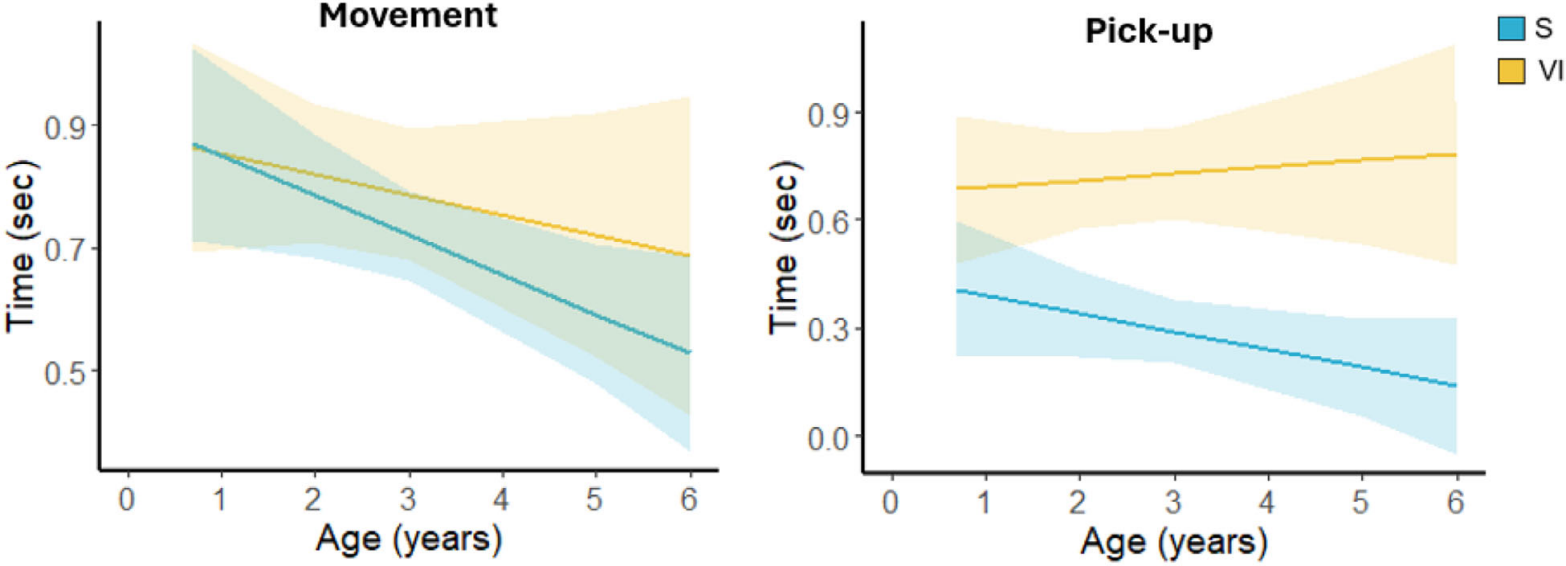
Relationship between age and time parameters. The graphs show the regression lines illustrating the relationship between age and time for Movement (on the left) and Pick‐up (on the right). The solid light‐blue line represents the best‐fit linear model for the sighted group, while the solid yellow line represents the VI group. The shaded areas around the regression lines indicate the 95% confidence intervals, reflecting the variability and uncertainty around the mean estimates for each group. For the Movement phase, there is a negative relationship between age and action time in both groups, with the sighted group showing a steep decline. In the pick‐up phase, the sighted children become faster with age.

In line with our hypothesis, we observed a main effect of Age on Movement time [*X* ^2^ (1) = 5.97, *p* = 0.01] with a reduction in Movement time with age, regardless of group (Figure 3, left). Moreover, we found a main effect of Group on Pick‐up time [*X* ^2^ (1) = 30.15, *p* < 0.001] with VI children significantly slower than the S peers, regardless of age (estimate = 0.45, SE = 0.08, *t*‐ratio = 5.63, df = 44.10, *p* < 0.001) (Figure 3, right). However, no significant interaction was found between Age and Group. We performed additional analyses to test any differences between groups independently of age, with consistent results (Figure S2). The means and standard deviations of the temporal parameters in both groups are reported in Table S7.

### 3.2 Reduced One‐hand Body Midline Crossing in Visually Impaired Children

**FIGURE 4:**
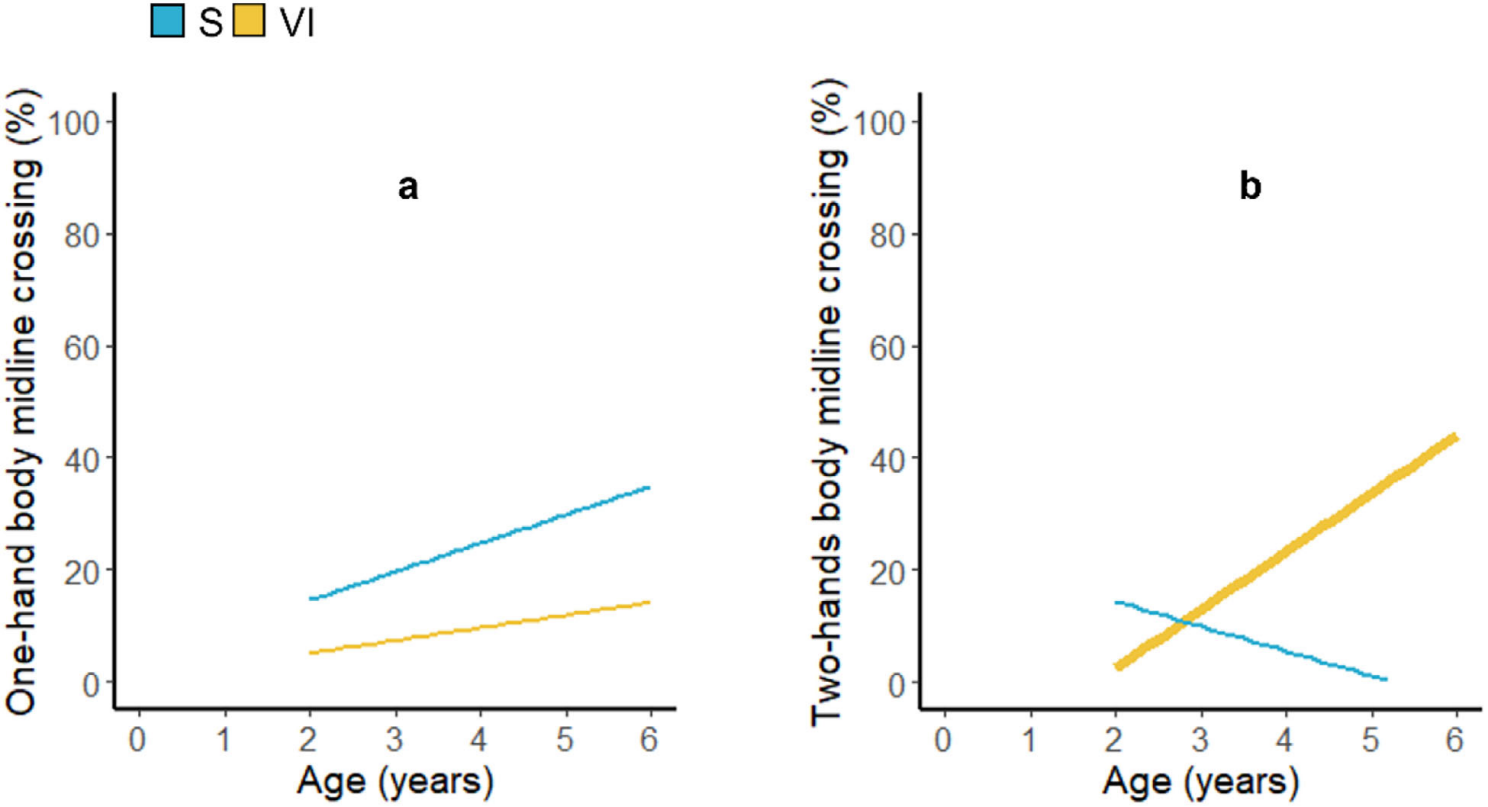
Relationship between age and (a) one‐hand and (b) two‐hands body midline crossing in the two groups Here we report the regression lines illustrating the relationship between age and body midline crossing, that is, the percentage of body midline crossing during reach‐to‐grasp for spheres placed at sides with (a) one hand and two hands (b). The solid light‐blue line represents the best‐fit linear model for the S group, while the solid yellow line the VI group. The thick lines in VI group indicate a significant relationship between age and two‐hands body midline crossing.

We found no significant Age × Group interaction on the percentage of body midline crossing with one hand (see Figure 4a) according to our hypothesis. However, we found a main effect of Group [*X* ^2^ (1) = 5.74, *p* = 0.021] with the S group showing a higher mean percentage compared to VI regardless of age (S: 20.24 ± 2.80; VI: 7.64 ± 4.22).We also observed a significant Age × Group interaction two‐hands bodymidline crossing [*X* ^2^ (1) = 11.37, *p* = 0.002], with increased percentage of two‐hands body midline crossing with age in the VI group, but not in the S children (age = 10.36, SE = 3.48, *t*‐ratio = 2.98, df = 43.00, *p* = 0.005) (Figure 4b). As for time parameters, we performed additional analyses to test any differences between groups independently of age, with consistent results (Figure S3).

**FIGURE 5:**
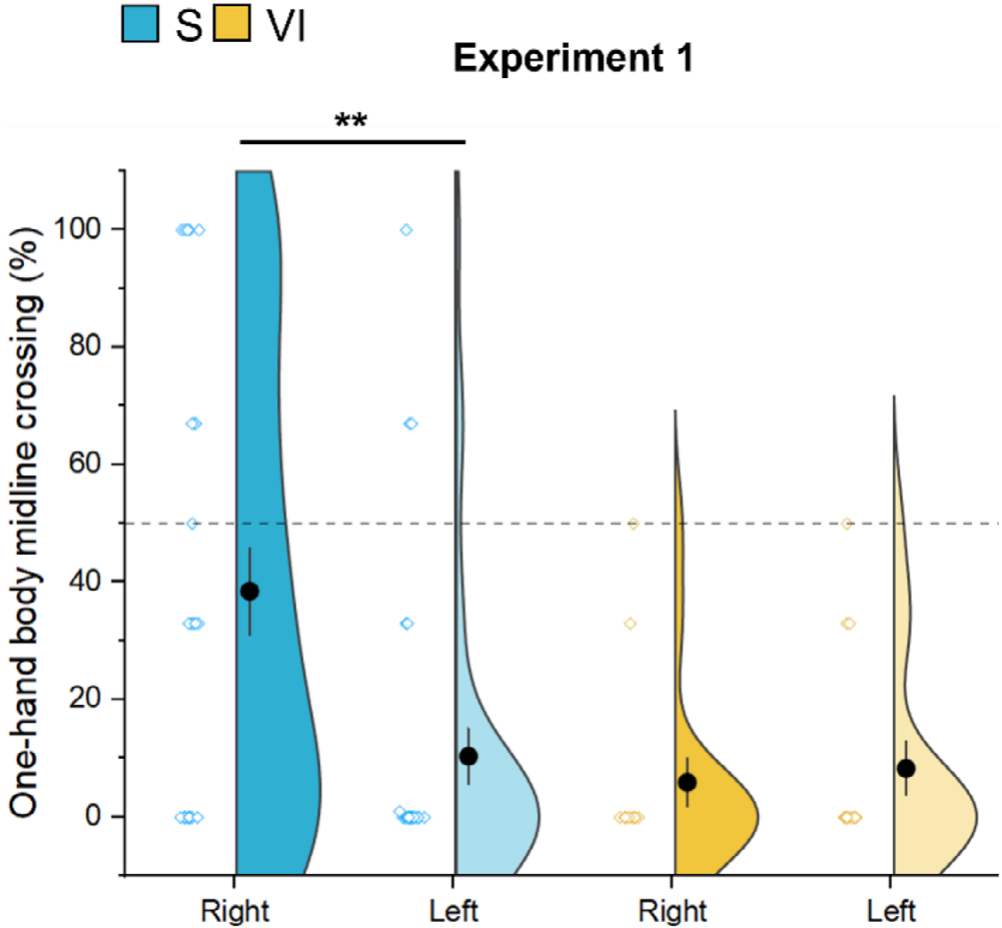
Comparison between right and left‐hand body midline crossing in the S and VI groups. The solid blue violin plot represents the right‐hand crossing, and the light blue the left‐hand crossing in the S group. The solid yellow violin plot represents the right‐hand crossing, and the light‐yellow the left‐hand crossing in the VI group. Each violin plot shows the distribution and variability of hand usage during body midline crossing. The colored dots are the subjects; the black dot at the center of each violin plot indicates the mean and the black line the standard error. A dashed horizontal line at 50% is included to aid visual comparison. **p < 0.01.

Finally, we observed a main effect of hand preference on one‐hand body midline crossing regardless of age [*X* ^2^ (1) = 7.36, *p* = 0.007] with a higher percentage of one‐hand body midline crossing with the right rather than the left hand in the S group only (estimate = −0.25, *t*‐ratio = −2.71, *p* = 0.01) (Figure 5).

The means and standard deviations of the motor parameters are reported in Tables S8–S9 and S10–S11.

## 4 General Discussion

In this study, we investigated for the first time the reach‐to‐grasp behavior in a sample of congenital VI children during their first six years of life and compared them to S peers. We adopted a multifaceted approach, exploring both temporal and motor behavior parameters involved in the ability to reach and grasp spheres of different dimensions. Here we report three key findings. First, visually impaired children require more time to Pick‐up the spheres than their S peers. Second, in our sample, VI children show a reduced percentage of one‐hand body midline crossing during reach‐to‐grasp for lateral spheres. They also exhibit an age‐related increase in body midline crossing particularly when using both hands. Third, the VI children do not show any hand preference compared to S children who preferably use the right during the body midline crossing to reach for lateral spheres hand. We discuss the implications of these results below.

### 4.1 Early Visual Impairment Influences the Pick‐Up But Not the Movement Phase During Reach‐to‐Grasp Task

In experiment 1, in line with our hypothesis, we found that VI children required more time to Pick‐up the spheres than S children. This suggests that visual experience in the first years of life plays a different role depending on the phase considered during the reach‐to‐grasp behavior, with greater influence on the Pick‐up rather than Movement phase. A possible explanation of this finding is that picking up an object requires the ability to extract knowledge about the object using its visual properties (e.g., shape and size), which is typically impaired in people with visual disabilities (Hasselt and Sisson 1987). Conversely, moving the hand toward an object primarily depends on extrinsic object properties, like spatial localization, and may not demand extensive visual knowledge about the object's features. Thus, early visual experience influences more the former (i.e., grip or intrinsic component), but not the latter (i.e., transport or extrinsic component). In other words, early visual experience helps in shaping the ability to effectively program object pick‐up based on its properties. Conversely, the time required to move toward the object using auditory feedback generated by its bumping on the table is not affected by early visual experience. This is also supported by neuroscientific studies showing distinct neural correlates underlying the transport (Movement) and grip (Pick‐up) components, including the superior parieto‐occipital sulcus and the anterior intraparietal sulcus, respectively. These areas are involved in processing both intrinsic and extrinsic object information separately, and in integrating them to effectively coordinate the reach‐to‐grasp action (Bosco et al. 2023; Monaco et al. 2015; Tunik et al. 2005).

Our results on time parameters align partially with those of Piller et al. (2023), who found differences in both time parameters between sighted children and patients after cataract removal. This discrepancy may be related to differences in the task setup. In particular, in our study, the task was performed in a visually adapted environment, that minimized visual input to ensure a fair comparison between groups. This setup also allowed us to understand the influence of early visual experience on the interaction between the child and the surrounding environment, relying on other sensory channels than vision. The absence of differences in Movement time between groups is consistent with findings by Gori et al. (2014), who demonstrated comparable performance between sighted and congenitally blind individuals in simple auditory spatial tasks, wherein participants were asked to localize a sound, in contrast with more complex task that required estimating its relative spatial position with respect to the position of two landmarks (Gori et al. 2014).

The distinct effect of early visual experience on the two reach‐to‐grasp phases is also in line with our results showing increased Movement than Pick‐up time in S children, and that Movement time decreases with age in both groups, with no difference between them. Thus, S children, drawing from their sensory‐motor experiences shaped by visual input, can quickly execute motor programs for picking up the sphere, likely because visual input enhances tactile perception by improving precision just before the fingers touch the object (Juravle et al. 2018). As children grow, their ability to use auditory feedback to locate and reach for the sphere improves, even in those with visual impairments. Despite the lack of visual experience, VI children improve in locating and moving toward objects, but they still struggle with adapting their hand to the sphere to perform a quick Pick‐up regardless of age.

### 4.2 Early Visual Impairment Is Associated With Reduced One‐Hand Body Midline Crossing in Children

In Experiment 1, we observed a higher percentage of one‐hand body midline crossing, but not two hands, among S children compared to VI children, regardless of age. A possible explanation of these findings is that VI children often experience difficulties in motor skills from infancy, affecting their postural control, balance, and gross motor abilities (Fazzi et al. 2011; Levtzion‐Korach et al. 2000; Prechtl et al. 2001). The absence of visual input also affects their ability to integrate information from multiple modalities, making difficult to understand their body's position in space leading to challenges in developing an egocentric perspective, particularly when crossing the body's midline to reach for lateral spheres (Martolini et al. 2021; Pasqualotto and Proulx 2012).

Moreover, visual experience appears to be crucial for developing hand preference. Specifically, in Experiments 1–2 S children exhibited higher percentage of body midline crossing with their right hand, whereas VI children did not. Consequently, hand preference may influence the overall tendency of S children to perform one‐hand body midline crossing when reaching for spheres placed at the side. In contrast, VI children did not adopt any consistent motor strategy, since no clear hand preference was observed and the percentage of body midline crossing occurred less frequently than that of S children.

We also found an increment in two‐hand body midline crossing with age in VI children but not in their sighted peers in both experiments. This might be because sighted children typically develop this ability around 5 months (van Hof et al. 2002). The minimum age of the S group is 1 year old, meaning that this skill is already developed in S children included in our study. Conversely, the increased percentage of two‐hands body midline crossing with age in VI children may reflect the delay in the development of this ability. Alternatively, due to the lack of visual input, it could be a learned functional strategy as they usually rely on tactile exploration provided by both hands to better understand objects' features. This is supported by some studies on adult participants showing that blind individuals benefit more from using both hands during tactile exploration and size representation of familiar objects compared to sighted peers (Guerreiro et al. 2015; Morash et al. 2014; Smith et al. 2005). Thus, VI children tend to use two hands for spatial interaction as they grow, but without demonstrating a clear hand preference during one‐handed body midline crossing. In contrast, S children showed similar pattern across in both experiments when interacting with larger spheres. Specifically, the data suggest that there has been no increase in the use of two hands when reaching for balls placed to the side and show a preference for using their right hand during one handed body midline crossing. We hypothesize that the balls used in the second experiment were likely not large enough to warrant using two hands for an effective Pick‐up. Therefore, sighted children could grasp them without difficulty using only one hand. In this regard, further investigation is needed to test our idea by increasing the diameter of the spheres.

### 4.3 Limitations

Several limitations emerge from our study; however, they can be addressed in future studies.

First, the number of trials planned for each participant is limited, with each sphere dimension being presented only once in each position. Given the young age of the children in our sample, we made this choice to avoid fatigue and maintain a good level of compliance, ensuring the collection of reliable data. As a result, we did not include position and size as factors to increase the statistical power of our data analysis. However, it would be interesting to add the number of trials for each position and sphere size in order to investigate whether these factors influence temporal and motor parameters of reach‐to‐grasp in cases of early visual impairment.

Second, it is important to note that the age range considered in this study is quite broad, and children undergo significant changes in various developmental areas during the first 6 years of life. Nevertheless, considering again the characteristics of the clinical population involved, it is essential to recognize that recruiting an adequate number of children in a younger age range is challenging. Indeed, children with congenital visual impairments often present with additional impairments (e.g., motor or cognitive). Additionally, these families require careful and sensitive support, making it not always feasible or appropriate to request their participation in research studies. Even so, to make more accurate speculation regarding the role of vision in the early years of life in reach‐to‐grasp skills, it would be still necessary to narrow the age range.

Fourth, we did not include a formal assessment of handedness. This decision was based on the preschool age of the participants, who had not yet developed consistent writing skills, which are often used as an indicator of hand dominance (Roszkowski et al. 1981).

Finally, it would be certainly necessary to refine the experimental procedure, including potential adjustments (e.g., increasing the number of digital video cameras and improving their location) to enhance video analysis capabilities. This optimization aims to facilitate the extraction of precise data such as the kinematic analysis of movement using appropriate software capable of deriving such information from video recordings.

## 5 Conclusions

To summarize, visually impaired children have longer Pick‐up time compared to their sighted peers during a reach‐to‐grasp task and tend to perform fewer one‐hand body midline crossing, without exhibiting a preference for either the right or left hand. Moreover, it seems that they learn at a later age to use both hands to grasp spheres placed to the side, even though the ball's size would allow for more efficient one‐handed strategies. Maybe, it could be a smart strategy for better exploring the objects. To conclude, this experimental approach allows us to investigate the reach‐to‐grasp skills in early life stages, thereby fostering interesting insights for the development of early scientific‐based rehabilitation protocols. Indeed, based on our results and the reliance on the sense of touch for object manipulation and recognition among VI children (Purpura et al. 2021), the functional use of two hands are aspects that should be considered during rehabilitation. Moreover, our results support the idea that early visual experience plays a fundamental and integral role in a child's overall development. It serves as an adaptive function by guiding and mediating other sensory inputs, helping children to represent the space around them, understand their body in motion, and interact with objects.

## Author Contributions

**Petri Stefania**: conceptualization, investigation, writing – original draft, writing – review and editing, methodology, formal analysis, data curation. **Riberto Martina**: writing – original draft, methodology, writing – review and editing, formal analysis, data curation. **Setti Walter**: writing – original draft, methodology, writing – review and editing, formal analysis, data curation. **Campus Claudio**: formal analysis, writing – review and editing, methodology, software. **Vitali Helene**: writing – review and editing, methodology. **Signorini Sabrina**: resources, conceptualization, writing – review and editing. **Tinelli Francesca**: resources, conceptualization, writing – review and editing. **Serafino Massimiliano**: resources, conceptualization, writing – review and editing. **Strazzer Sandra**: conceptualization, resources, writing – review and editing. **Giammari Giuseppina**: conceptualization, resources, writing – review and editing. **Cocchi Elena**: conceptualization, resources, writing – review and editing. **Gori Monica**: funding acquisition, conceptualization, investigation, writing – original draft, writing – review and editing, methodology, project administration, supervision, resources, visualization.

## Ethics Statement

This study was approved by the Ethics Committees of centers participating in the study (Tuscany Region Ethics Committee—Paediatric, Liguria Region Ethics Committee, IRCCS E.Medea Ethics Committee, IRCCS Mondino Foundation Ethics Committee).

## Conflicts of Interest

The authors declare no conflicts of interest.

## Supporting information




**Supporting File 1**: desc70067‐sup‐0001‐SuppMat.docx

## Data Availability

The data are available in Zenodo: https://doi.org/10.5281/zenodo.14677793.
