# Supplementary material for: Impact of Congenital Visual Impairment on Early‐Life Exploration: Behavioral Analysis of Temporal and Motor Parameters During a Reach‐to‐Grasp Playful Task
Source: Dev Sci. 2025 Aug 24;28(5):e70067. doi: 10.1111/desc.70067 (PMC12375338; doi:10.1111/desc.70067)
Supplement: Supplementary file 1 — Supporting File 1: desc70067‐sup‐0001‐SuppMat.docx [file DESC-28-e70067-s001.docx]

**Supplementary materials**

| Children | Gender | Age (years) | Cause of Visual Impairment | Visual Acuity (LogMar) | Experiment  Performed |
| --- | --- | --- | --- | --- | --- |
| VI01 | Female | 1,8 | Anophtalmia | >1.0 | 1 |
| VI02 | Female | 2,9 | Retinal dystrophy | >1.0 | 1 |
| VI03 | Male | 0,7 | Congenital cataract | 0.82 | 1 |
| VI04 | Male | 1,3 | Ocular albinism | 1.0 | 1 |
| VI05 | Female | 5,0 | Leber congenital amaurosis | na | 1 |
| VI06 | Male | 4,1 | Nystagmus | 1.0 | 1 |
| VI07 | Male | 1,7 | Nystagmus | 0.82 | 1 |
| VI08 | Male | 2,3 | Ocular albinism | >1.0 | 1 and 2 |
| VI09 | Male | 6 | Retinal dystrophy | na | 1 and 2 |
| VI10 | Male | 1,3 | Retinal dystrophy | >1.0 | 1 and 2 |
| VI11 | Male | 2,3 | Congenital cataract | 0.7 | 1 |
| VI12 | Female | 1,3 | Ocular albinism | na | 1 |
| VI13 | Male | 3,3 | Leber congenital amaurosis | >1.0 | 1 and 2 |
| VI14 | Male | 2 | Optic nerve hypoplasia | >1.0 | 1 and 2 |
| VI15 | Female | 3,8 | Leber congenital amaurosis | >1.0 | 1 and 2 |
| S01 | Female | 3,0 | - | - | 1 |
| S02 | Female | 2,3 | - | - | 1 |
| S03 | Male | 2,0 | - | - | 1 |
| S04 | Male | 2,8 | - | - | 1 |
| S05 | Male | 2,9 | - | - | 1 |
| S06 | Female | 5,5 | - | - | 1 |
| S07 | Female | 1,1 | - | - | 1 |
| S08 | Female | 5,2 | - | - | 1 |
| S09 | Female | 3,4 | - | - | 1 |
| S10 | Male | 4,8 | - | - | 1 |
| S11 | Female | 4,0 | - | - | 1 |
| S12 | Female | 2,7 | - | - | 1 |
| S13 | Male | 5,1 | - | - | 1 |
| S14 | Male | 2,2 | - | - | 1 |
| S15 | Male | 1,2 | - | - | 1 and 2 |
| S16 | Female | 2,4 | - | - | 1 and 2 |
| S17 | Female | 5,8 | - | - | 1 and 2 |
| S18 | Female | 2,0 | - | - | 1 and 2 |
| S19 | Male | 3,3 | - | - | 1 |
| S20 | Female | 5,4 | - | - | 1 and 2 |
| S21 | Male | 2,9 | - | - | 1 |
| S22 | Female | 2,9 | - | - | 1 |
| S23 | Female | 5,3 | - | - | 1 and 2 |
| S24 | Male | 3,4 | - | - | 1 and 2 |
| S25 | Female | 4,7 | - | - | 1 and 2 |
| S26 | Male | 4,0 | - | - | 1 and 2 |
| S27 | Male | 3,4 | - | - | 1 and 2 |
| S28 | Male | 4,1 | - | - | 1 and 2 |
| S29 | Female | 3,1 | - | - | 1 |
| S30 | Male | 1,8 | - | - | 1 |
| S31 | Female | 1,3 | - | - | 1 |
| S32 | Female | 2,7 | - | - | 1 |

Table S1. Clinical and demographic information of visually impaired and sighted children. Abbreviations: VI, visually impaired; S, sighted; na, not available.

**Experiment 2: materials and methods**

A subgroup of children who participated in Experiment 1 also took part in Experiment 2. Specifically, six VI (6 males, mean age 3.0 ± 1.5 y.o) and eleven S (7 females, mean age 3.7 ± 1.8 y.o) children were included in the subsample.

In this exploratory experiment, we introduced two additional larger spheres (10 cm and 12 cm in diameter), resulting in five total stimulus sizes: small (3cm), medium (5cm), big1 (8cm), big2 (10cm) and big3 (12cm) (Figure S1). To present the stimuli, we randomized the dimensions Experiment 2: small, medium, big1, big 2, big3) and the positions (e.g., left, right, center).

Experiment 2 lasted about fifteen minutes (fifteen trials in total).


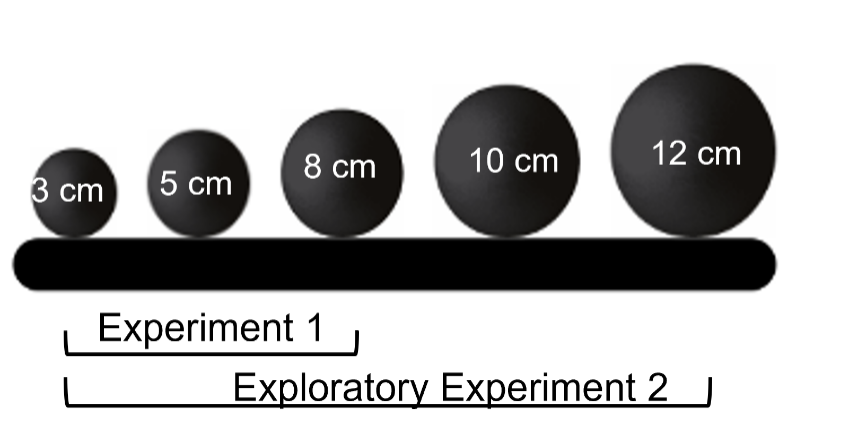
Despite, the experiment 1, in which both motor and temporal parameters were analysed, only motor parameters were analyzed in this second experiment.

Figure S1: Experimental stimuli. a) Black spheres with different diameter sizes used in the experiment 1 and 2: 3cm, 5cm, 8cm, 10cm and 12cm.

| **Trial** | **Size** | **Position** | **Experiment** |
| --- | --- | --- | --- |
| 1 | Small | Centre | 1 and 2 |
| 2 | Big1 | Left | 1 and 2 |
| 3 | Small | Right | 1 and 2 |
| 4 | Big1 | Right | 1 and 2 |
| 5 | Big1 | Centre | 1 and 2 |
| 6 | Small | Left | 1 and 2 |
| 7 | Medium | Centre | 1 and 2 |
| 8 | Medium | Right | 1 and 2 |
| 9 | Medium | Left | 1 and 2 |
| 10 | Big2 | Centre | 2 |
| 11 | Big3 | Left | 2 |
| 12 | Big2 | Right | 2 |
| 13 | Big3 | Centre | 2 |
| 14 | Big3 | Right | 2 |
| 15 | Big2 | left | 2 |

Table S2. Order of sphere presentation during Experiments 1 and 2. Here, we report the order of presentation trial by trial, which was the same for each child. Specifically, we randomized the dimensions (e.g., Experiment 1: small, medium, big1; Experiment 2: small, medium, big1, big 2, big3) and the positions (e.g., left, right, center).

|  | | **Number of Pick-up attempts across trials** | | | | | | | | |
| --- | --- | --- | --- | --- | --- | --- | --- | --- | --- | --- |
| Children | Number of successful Pick-up | 1 | 2 | 3 | 4 | 5 | 6 | 7 | 8 | 9 |
| VI01 | 9/9 | 1 | 2 | 1 | 1 | 1 | 1 | 1 | 2 | 1 |
| VI02 | 6/9 | 1 | 1 | 1 | / | / | 1 | 1 | 1 | / |
| VI03 | 8/9 | 1 | 2 | 1 | 1 | 1 | 1 | 1 | 1 | / |
| VI04 | 9/9 | 1 | 2 | 1 | 1 | 2 | 1 | 1 | 2 | 1 |
| VI05 | 4/9 | 1 | / | / | / | 1 | 1 | / | / | 1 |
| VI06 | 9/9 | 1 | 1 | 1 | 1 | 1 | 1 | 1 | 1 | 1 |
| VI07 | 9/9 | 1 | 1 | 1 | 1 | 1 | 1 | 2 | 2 | 1 |
| VI08 | 9/9 | 1 | 1 | 1 | 1 | 1 | 1 | 1 | 1 | 2 |
| VI09 | 9/9 | 1 | 1 | 1 | 1 | 1 | 1 | 1 | 1 | 1 |
| VI10 | 8/9 | 1 | / | 1 | 1 | 1 | 1 | 1 | 1 | 1 |
| VI11 | 8/9 | 1 | 1 | 1 | / | 1 | 1 | 1 | 1 | 1 |
| VI12 | 9/9 | 1 | 1 | 1 | 1 | 1 | 1 | 1 | 1 | 1 |
| VI13 | 9/9 | 1 | 1 | 1 | 1 | 1 | 1 | 1 | 1 | 1 |
| VI14 | 9/9 | 1 | 1 | 1 | 1 | 1 | 1 | 1 | 1 | 1 |
| VI15 | 9/9 | 1 | 1 | 1 | 1 | 1 | 1 | 1 | 1 | 1 |

Table S3. Number of Pick-up attempts per trial for each child in the visually impaired (VI) group. The table reports, for each participant, the number of attempts needed to achieve a successful Pick-up, as agreed upon by both raters.

|  | | **Number of Pick-up attempts across trials** | | | | | | | | |
| --- | --- | --- | --- | --- | --- | --- | --- | --- | --- | --- |
| Children | Number of successful Pick-up | 1 | 2 | 3 | 4 | 5 | 6 | 7 | 8 | 9 |
| S01 | 8/9 | / | 1 | 2 | 1 | 2 | 1 | 1 | 2 | 1 |
| S02 | 9/9 | 1 | 1 | 1 | 1 | 1 | 1 | 1 | 1 | 1 |
| S03 | 9/9 | 1 | 1 | 1 | 1 | 1 | 1 | 1 | 1 | 1 |
| S04 | 9/9 | 1 | 1 | 1 | 1 | 1 | 1 | 1 | 1 | 1 |
| S05 | 9/9 | 1 | 1 | 1 | 1 | 1 | 1 | 1 | 1 | 1 |
| S06 | 9/9 | 1 | 1 | 1 | 1 | 1 | 1 | 1 | 1 | 1 |
| S07 | 8/9 | / | 1 | 1 | 1 | 1 | 1 | 1 | 1 | 1 |
| S08 | 8/9 | 1 | / | 1 | 1 | 1 | 1 | 1 | 1 | 1 |
| S09 | 9/9 | 1 | 1 | 1 | 1 | 1 | 1 | 1 | 1 | 1 |
| S10 | 9/9 | 1 | 1 | 1 | 1 | 1 | 1 | 1 | 1 | 1 |
| S11 | 9/9 | 1 | 1 | 1 | 1 | 1 | 1 | 1 | 1 | 1 |
| S12 | 8/9 | 1 | / | 1 | 1 | 1 | 1 | 1 | 1 | 1 |
| S13 | 9/9 | 1 | 1 | 1 | 1 | 1 | 1 | 1 | 1 | 1 |
| S14 | 9/9 | 1 | 1 | 1 | 1 | 1 | 1 | 1 | 1 | 1 |
| S15 | 9/9 | 1 | 1 | 1 | 1 | 1 | 1 | 1 | 1 | 1 |
| S16 | 9/9 | 1 | 1 | 1 | 1 | 1 | 1 | 1 | 1 | 1 |
| S17 | 9/9 | 1 | 1 | 1 | 1 | 1 | 1 | 1 | 1 | 1 |
| S18 | 9/9 | 1 | 1 | 1 | 1 | 1 | 1 | 1 | 1 | 1 |
| S19 | 8/9 | 1 | 1 | 1 | 1 | 1 | / | 1 | 1 | 1 |
| S20 | 9/9 | 1 | 1 | 1 | 1 | 1 | 1 | 1 | 1 | 1 |
| S21 | 9/9 | 1 | 1 | 1 | 1 | 1 | 1 | 1 | 1 | 1 |
| S22 | 9/9 | 1 | 1 | 1 | 1 | 1 | 1 | 1 | 1 | 1 |
| S23 | 9/9 | 1 | 1 | 1 | 1 | 1 | 1 | 1 | 1 | 1 |
| S24 | 9/9 | 1 | 1 | 1 | 1 | 1 | 1 | 1 | 1 | 1 |
| S25 | 9/9 | 1 | 1 | 1 | 1 | 1 | 1 | 1 | 1 | 1 |
| S26 | 9/9 | 1 | 1 | 1 | 1 | 1 | 1 | 1 | 1 | 1 |
| S27 | 9/9 | 1 | 1 | 1 | 1 | 1 | 1 | 1 | 1 | 1 |
| S28 | 9/9 | 1 | 1 | 1 | 1 | 1 | 1 | 1 | 1 | 1 |
| S29 | 9/9 | 1 | 1 | 1 | 1 | 1 | 1 | 1 | 1 | 1 |
| S30 | 8/9 | 1 | 1 | 1 | 1 | / | 1 | 1 | 1 | 1 |
| S31 | 9/9 | 1 | 1 | 1 | 1 | 1 | 1 | 1 | 2 | 1 |
| S32 | 9/9 | 1 | 1 | 1 | 1 | 1 | 1 | 1 | 1 | 1 |

Table S4. Number of Pick-up attempts per trial for each child in the sighted (S) group. The table reports, for each participant, the number of attempts needed to achieve a successful Pick-up, as agreed upon by both raters.

A Wilcoxon rank-sum test indicated no significant difference in the number of successful pick-up between VI and S groups (W=281.00, p=0.21).

|  | | **Number of Pick-up attempts across trials** | | | | | | | | | | | | | | |
| --- | --- | --- | --- | --- | --- | --- | --- | --- | --- | --- | --- | --- | --- | --- | --- | --- |
| Children | N° of successful Pick-up | 1 | 2 | 3 | 4 | 5 | 6 | 7 | 8 | 9 | 10 | 11 | 12 | 13 | 14 | 15 |
| VI08 | 14/15 | 1 | 1 | 1 | 1 | 1 | 1 | 1 | 1 | 2 | 1 | 1 | 1 | / | 1 | 2 |
| VI09 | 15/15 | 1 | 1 | 1 | 1 | 1 | 1 | 1 | 1 | 1 | 1 | 1 | 1 | 1 | 1 | 1 |
| VI10 | 12/15 | 1 | / | 1 | 1 | 1 | 1 | 1 | 1 | 1 | 1 | / | 2 | 2 | / | 1 |
| VI13 | 14/15 | 1 | 1 | 1 | 1 | 1 | 1 | 1 | 1 | 1 | 1 | / | 1 | 1 | 1 | 1 |
| VI14 | 13/15 | 1 | 1 | 1 | 1 | 1 | 1 | 1 | 1 | 1 | 1 | / | 1 | 1 | / | 1 |
| VI15 | 15/15 | 1 | 1 | 1 | 1 | 1 | 1 | 1 | 1 | 1 | 1 | 1 | 1 | 1 | 1 | 1 |

Table S5. Number of Pick-up attempts per trial (Experiment 2) for each child in the visually impaired (VI) group. The table reports, for each participant, the number of attempts needed to achieve a successful Pick-up, as agreed upon by both raters.

|  | | **Number of Pick-up attempts across trials** | | | | | | | | | | | | | | |
| --- | --- | --- | --- | --- | --- | --- | --- | --- | --- | --- | --- | --- | --- | --- | --- | --- |
| Children | N° of successful Pick-up | 1 | 2 | 3 | 4 | 5 | 6 | 7 | 8 | 9 | 10 | 11 | 12 | 13 | 14 | 15 |
| S15 | 13/15 | 1 | 1 | 1 | 1 | 1 | 1 | 1 | 1 | 1 | 1 | 1 | / | 1 | / | 2 |
| S16 | 14/15 | 1 | 1 | 1 | 1 | 1 | 1 | 1 | 1 | 1 | 1 | 1 | / | 1 | 1 | 1 |
| S17 | 15/15 | 1 | 1 | 1 | 1 | 1 | 1 | 1 | 1 | 1 | 1 | 1 | 1 | 1 | 1 | 1 |
| S18 | 14/15 | 1 | 1 | 1 | 1 | 1 | 1 | 1 | 1 | 1 | 1 | 1 | 1 | 1 | / | 1 |
| S20 | 15/15 | 1 | 1 | 1 | 1 | 1 | 1 | 1 | 1 | 1 | 1 | 1 | 1 | 1 | 1 | 1 |
| S23 | 15/15 | 1 | 1 | 1 | 1 | 1 | 1 | 1 | 1 | 1 | 1 | 1 | 1 | 1 | 1 | 1 |
| S24 | 15/15 | 1 | 1 | 1 | 1 | 1 | 1 | 1 | 1 | 1 | 1 | 2 | 1 | 1 | 1 | 1 |
| S25 | 15/15 | 1 | 1 | 1 | 1 | 1 | 1 | 1 | 1 | 1 | 1 | 1 | 1 | 1 | 1 | 1 |
| S26 | 15/15 | 1 | 1 | 1 | 1 | 1 | 1 | 1 | 1 | 1 | 1 | 1 | 1 | 1 | 1 | 1 |
| S27 | 15/15 | 1 | 1 | 1 | 1 | 1 | 1 | 1 | 1 | 1 | 1 | 1 | 1 | 1 | 1 | 1 |
| S28 | 15/15 | 1 | 1 | 1 | 1 | 1 | 1 | 1 | 1 | 1 | 1 | 1 | 2 | 1 | 1 | 1 |

Table S6. Number of Pick-up attempts per trial (Experiment 2) for each child in the sighted (S) group. The table reports, for each participant, the number of attempts needed to achieve a successful Pick-up, as agreed upon by both raters.

A Wilcoxon rank-sum test indicated no significant difference in the number of successful pick-up between VI and S groups (W=28.00, p=0.77).

##### ***Results: time parameters (only exp. 1)***

##### As shown in Figure S2, we found a significant Action x Group interaction [X ^2^ (1) = 47.19, p<0.001], with higher Pick-up time in VI than in S children (estimate= 0.44, SE=0.06, t-ratio=7.15, df=63.30, p<0.001) and no differences in Movement time between groups (estimate= -0.08, SE= 0.06, t-ratio=-1.23, df=64.10, p=1.000). Also, S children are faster in Pick-up than in Movement (estimate= -0.43, SE=0.03, t-ratio=-16.01, p<0.001), while no significant differences between Pick-up and Movement were observed in the VI group (estimate= -0.10, SE=0.04, t-ratio=-2.39, p=0.104).

| **Group** | **Variable** | **Mean (sec)** | **Standard Deviation** |
| --- | --- | --- | --- |
| Visually Impaired | Movement Time | 0.80 | 0.41 |
| Visually Impaired | Pick Up Time | 0.70 | 0.47 |
| Sighted | Movement Time | 0.70 | 0.29 |
| Sighted | Pick Up Time | 0.27 | 0.28 |


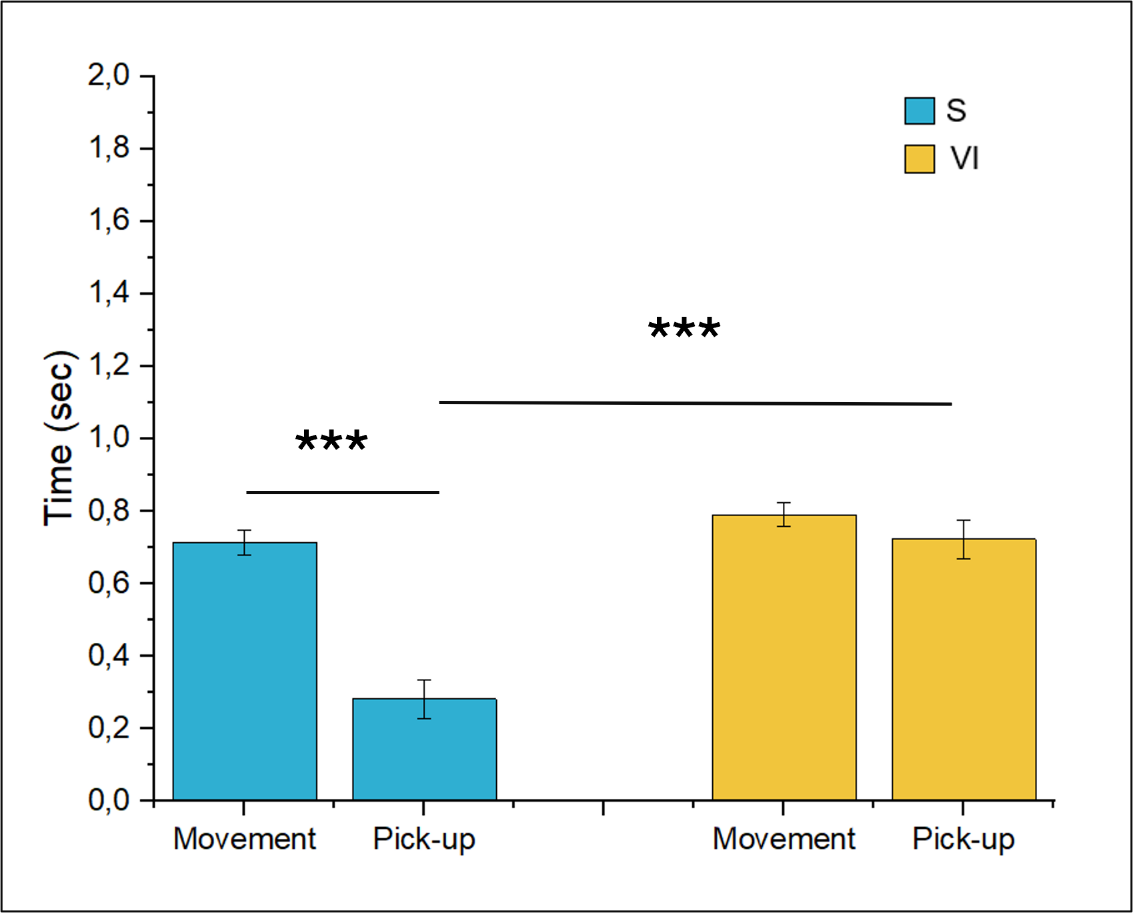
Table S7. Means and Standard Deviation values of Time parameters Experiment 1.

**Experiment 1**

Figure S2. Differences between groups in reach-to-grasp time. Here, we report the average time (in seconds) during movement and pick-up for each group. Blue colors denote sighted children, yellow colors visually impaired children. Error bars indicate ±2 SEM. ***, p<0.001.

##### ***Results: differences between groups in body-midline crossing (exp 1-2)***

Experiment 2 replicated all the findings of experiment 1 on motor parameters, except for the difference between groups in the percentage of one-hand body midline crossing. As shown in Figure S3, we found a significant difference between groups in the percentage of one-hand body midline crossing [F(1,45) = 8.26, p=0.006], with a higher percentage in the S group in Experiment 1, but not in Experiment 2 (Figure S3 a). However, we did not observe any significant difference between groups in two-hands body midline crossing in both Experiments 1 and 2 (Figure S3 b).

| **Group** | **Variable** | **Percentage (%)** | **Standard Deviation** |
| --- | --- | --- | --- |
| Visually Impaired | One-H body midline crossing | 6.60 | 9.80 |
| Visually Impaired | Two-H body midline crossing | 9.33 | 24.90 |
| Sighted | One-H body midline crossing | 21.30 | 18.60 |
| Sighted | Two-H body midline crossing | 8.44 | 20.80 |

Table S8. Means and Standard Deviation values of Motor parameters (One-Two hand body midline crossing) Experiment 1.

| **Group** | **Variable** | **Percentage (%)** | **Standard Deviation** |
| --- | --- | --- | --- |
| Visually Impaired | One-H body midline crossing | 10.70 | 13.40 |
| Visually Impaired | Two-H body midline crossing | 23.10 | 35.10 |
| Sighted | One-H body midline crossing | 25.60 | 21.70 |
| Sighted | Two-H body midline crossing | 21.30 | 31.40 |


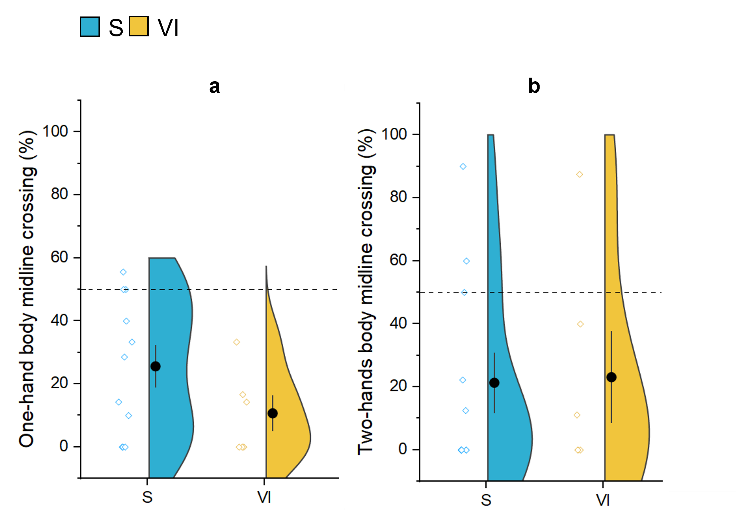
Table S9. Means and Standard Deviation values of Motor parameters (One-Two hand body midline crossing) Experiment 2.

Figure S3. Differences between groups in (a) one-hand and (b) two-hands body midline crossing in Experiments 1 (left) and 2 (right). Each violin plot shows the distribution and variability of the percentage of body midline crossing (one-hand and two-hands) for each group. Blue color represents the S group, while yellow color the VI group. The colored dots are the subjects; the black dot of each plot indicates the median and the vertical black line the standard error. A dashed horizontal line at 50% is included to aid visual comparison. **, p<0.01.


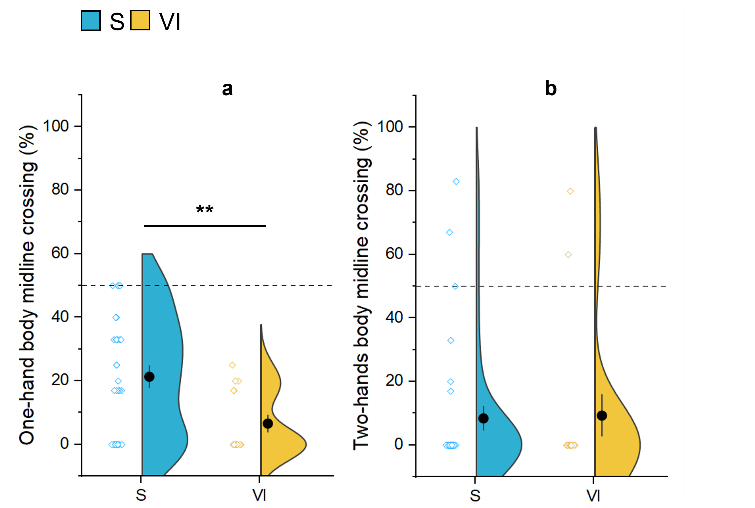


**Experiment 1**

**Experiment 2**

##### ***Results: effect of age on body-midline crossing (exp 2)***

#####
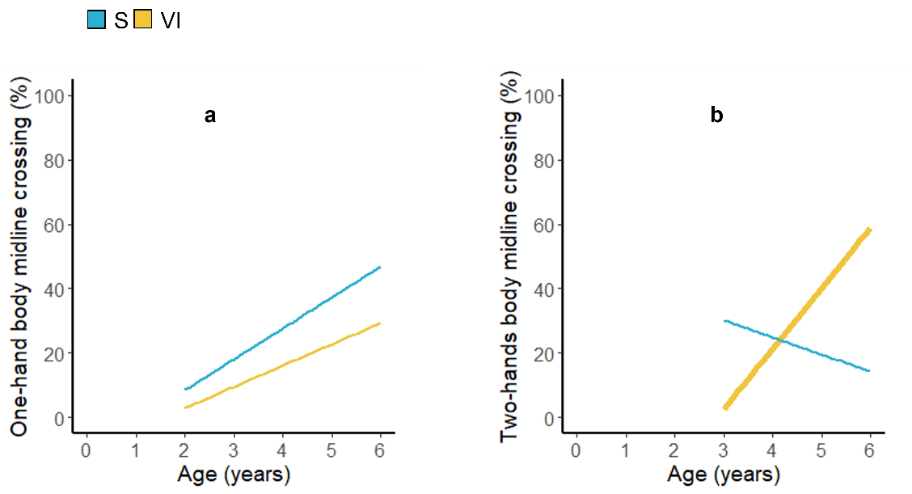
Then, we observed no significant Age x Group interaction on the percentage of body midline crossing with one hand (see Figure S4 a) according to our hypothesis. We also observed a significant Age x Group interaction two-hands bodymidline crossing [*X* ^2^ (1) = 11.17, p=0.005], with increased percentage of two-hands body midline crossing with age in the VI group, but not in the S children (age = 18.70, SE=6.60, t-ratio = 2.82, df=13.00, p=0.01) (Figure S4 b).

**Experiment 2**

Figure S4. **Experiment 2:** relationship between age and (a) one-hand and (b) two-hands body midline crossing in the two groups. Here we report the regression lines illustrating the relationship between age and body midline crossing, that is, the percentage of body midline crossing during reach-to-grasp for spheres placed at sides with (a) one hand and (b) with two hands. The solid light-blue line represents the best-fit linear model for the S group, while the solid yellow line the VI group. The thick lines in VI group indicate a significant relationship between age and two-hands body midline crossing.

##### ***Results: differences between groups in hand preference (exp 2)***

Finally, we replicated all the findings of experiment 1 founding a main effect of hand preference on one-hand body midline crossing regardless of age [*X* ^2^ (1) = 12.54, p<0.001] with a higher percentage of one-hand body midline crossing with the right rather than the left hand in the S group only (estimate= -0.51, t-ratio = -3.54, p=0.01) (Figure S5).

| **Group** | **Variable** | **Percentage (%)** | **Standard Deviation** |
| --- | --- | --- | --- |
| Visually Impaired | Right Hand | 5.95 | 15.50 |
| Visually Impaired | Left Hand | 8.33 | 17.00 |
| Sighted | Right Hand | 38.50 | 39.60 |
| Sighted | Left Hand | 13.80 | 30.20 |

Table S10. Means and Standard Deviation values of Motor parameters (Hand preference) Experiment 1.

| **Group** | **Variable** | **Percentage (%)** | **Standard Deviation** |
| --- | --- | --- | --- |
| Visually Impaired | Right Hand | 13.90 | 22.20 |
| Visually Impaired | Left Hand | 4.17 | 10.20 |
| Sighted | Right Hand | 58.30 | 40.40 |
| Sighted | Left Hand | 7.78 | 17.20 |

Table S11. Means and Standard Deviation values of Motor parameters (Hand preference) Experiment 2.

Figure S5. Experiments 2: Comparison between right and left-hand body midline crossing in the S and VI groups. The solid blue violin plot represents the right-hand crossing, and the light blue the left-hand crossing in the S group. The solid yellow violin plot represents the right-hand crossing, and the light-yellow the left-hand crossing in the VI group. Each violin plot shows the distribution and variability of hand usage during body midline crossing. The colored dots are the subjects; the black dot at the center of each violin plot indicates the mean and the black line the standard error. A dashed horizontal line at 50% is included to aid visual comparison. **, p<0.01.


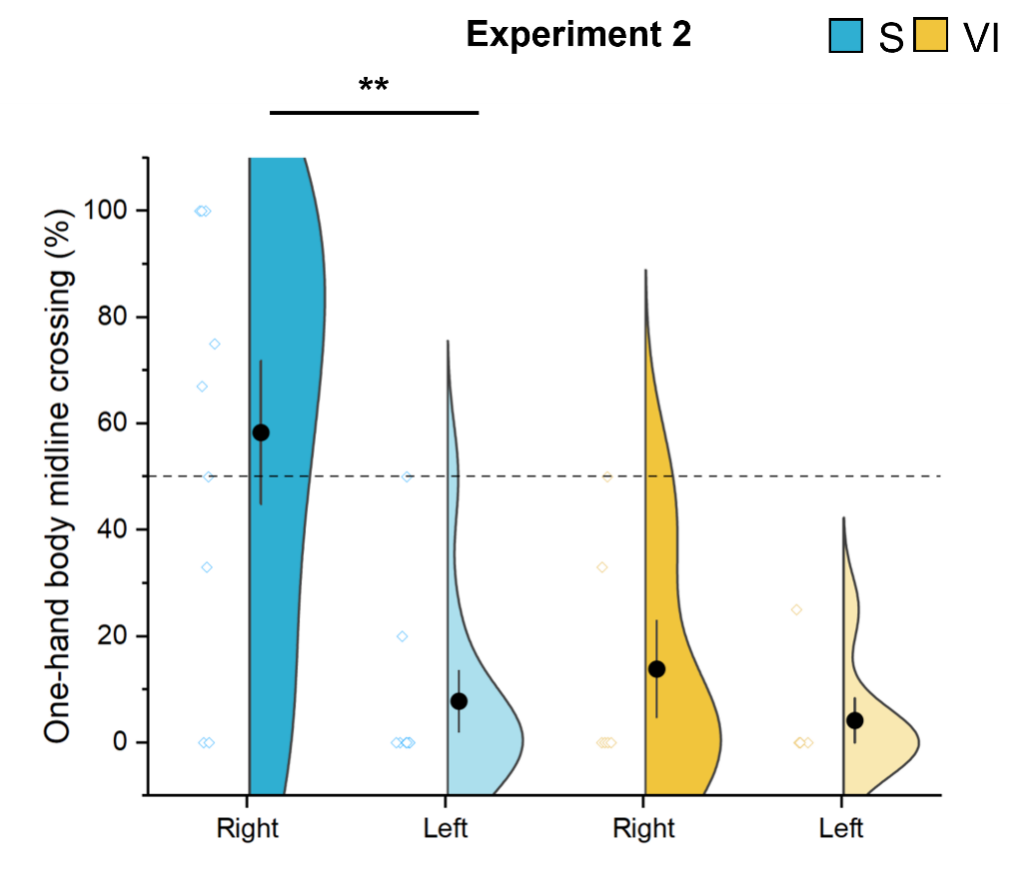


***Results:*** ***effect of Biological sex on body midline crossing (exp1-2)***

Experiment 1:

We conducted a LM with the percentage of one-hand body midline crossing as dependent variable, Group, Age and Biological Sex as predictors. We found no significant main effect of the Biological Sex [*X* ^2^ (1) = 0.29, p=0.592] nor interaction between Biological Sex and age [*X* ^2^ (1) = 0.73, p=0.399], group [*X* ^2^ (1) = 0.67, p=0.417] and age and group [*X* ^2^ (1) = 0.08 p=0.780] on the percentage of body midline crossing with one hand. Instead, we found a significant main effect of age [*X* ^2^ (1) = 5.72, p=0.022] and group [*X* ^2^ (1) = 5.99, p=0.020] on the percentage of body midline crossing with one hand. Model comparison based on Akaice Information Criterion (AIC) indicated that the model without Biological Sex provided a better fit to the data (AIC=397.63) than the model including it (AIC=403.75).

The same approach was used with the two-hands body midline crossing parameter. We found no significant main effect of the Biological Sex [*X* ^2^ (1) = 0.81, p=0.373] or interaction between Biological Sex and age [*X* ^2^ (1) = 0.05, p=0.830], group [*X* ^2^ (1) = 0.29, p=0.594] and age and group [*X* ^2^ (1) = 1.40 p=0.244] on the percentage of body midline crossing with one hand. Instead, we found an AgexGroup significant interaction [*X* ^2^ (1) = 9.52 p=0.004]. Model comparison based on Akaice Information Criterion (AIC) indicated that the model without Biological Sex provided a better fit to the data (AIC=421.29) than the model including it (AIC=426.35). It was not possible to include Biological Sex as a fixed effect in the model of Experiment 2 because all subjects in the VI group were male.
